# Supplementary material for: Negative mood affects the expression of negative but not positive emotions in mice
Source: Proc Biol Sci. 2020 Aug 26;287(1933):20201636. doi: 10.1098/rspb.2020.1636 (PMC7482280; doi:10.1098/rspb.2020.1636)
Supplement: Supplementary Materials [file rspb20201636supp1.docx]

**Supplementary Materials**

**“Negative mood affects the expression of negative but not positive emotions in mice”**

**Authors: Jasmine M Clarkson, Matthew C Leach, Paul A Flecknell, Candy Rowe**

**Proceedings of the Royal Society B**

**Main article DOI: 10.1098/rspb.2020.1636**

- 1. **Statistical analyses conducted for datasets reported in the main text.**

Table S1: Table detailing statistical analyses conducted on each dataset reported in the main text.

| **Data** | **Dependent Variable** | **Statistical Test** | **Factor(s)** | **Unit** | **Sample Size** |
| --- | --- | --- | --- | --- | --- |
| Behavioural and physiological measures of affective state | Voluntary Interaction: Percentage of time spent interacting | Generalised Linear Mixed Model (GLMM) with gamma distribution | Between subject factor: Handling method (2 levels) Within-subject factors: day (5 levels), time (2 levels: pre or post handling) | Cage | n=16 tail handled  n=16 tunnel handled |
|  | Elevated Plus Maze: Duration on open arms of elevated plus maze; Number of open arm entries; Number of protected stretch attend postures | Independent t-test | Handling method (2 levels) | Mouse | n=28 tail handled  n=29 tunnel handled |
|  | Open Field: Duration in centre of open field; Frequency in the centre | Independent t-test | Handling method (2 levels) | Mouse | n=32 tail handled  n=32 tunnel handled |
|  | Sucrose consumption; Lick cluster size  (pooled across the last four pre-shift trials) | General Linear Model (GLM) | Handling method (2 levels); Sucrose concentration (2 levels) | Mouse | n=32 tail handled  n=32 tunnel handled |
|  | Adrenal weight | Independent t-test | Handling method (2 levels) | Mouse | n=16 tail handled  n=16 tunnel handled |
|  | Sucrose consumption for control mice at the post-shift phase  (with post-shift phase as a factor) | Linear Mixed Model (LMM) | Between subject factor: Handling method (2 levels), Sucrose concentration (2 levels)  Within subject factor: Post-shift phase (2 levels) | Mouse | n=16 tail handled  n=16 tunnel handled  (control mice only) |
| Successive Negative and Positive Contrast effects  *Ran separately for SNC and SPC contrast conditions | Lick Cluster Size  (500ms criterion -at the two post-shift periods; see supplementary materials for additional criteria) | Linear Mixed Model (LMM) | Between subject factor: Handling method (2 levels), Contrast condition (2 levels)  Within subject factor: Post-shift phase (2 levels) | Mouse | n=16 (SPC)  n=16 (SPC controls)  n=16 (SNC)  n=16 (SNC controls) |

**1.2 Full results of the tests establishing the differences in affective state between tail and tunnel handed mice (see also Figure 2)**

*1.2.1 Voluntary interaction tests during the affective state manipulation (days 1, 5 and 9)*

A full factorial GLMM was carried out on the time spent interacting with handler, with handling method, day and time as main factors (Table S2).

Table S2: Full statistical results from the Generalised Linear Mixed Model (GLMM) conducted on the percentage of time spent voluntarily interacting with the handler on days 1, 5 and 9.

| **Factor** | ***χ*^2^** | **p value** |
| --- | --- | --- |
| Handling method  Day  Time (pre or post handling)  Handling method x Day  Handling method x Time  Day x Time  Handling method x Day x Time | 62.13  25.31  1.39  47.25  29.66  49.89  29.42 | 3.21^e-15^ ***  3.20^e-6^ ***  0.239 ns  1.35^e-9^ ***  1.624^e-6^ ***  3.81^e-10^ ***  4.084^e-7^ *** |

As outlined in the main text (see also Figure 2A), we found a main effect of handling method: tail handled mice spent significantly less time interacting with the handler than mice handled using a tunnel (***χ***^2^=62.13, p<0.001; Table S2). However, we also found other main effects and interactions. We also found that the timings of these tests were also important: there was a significant main effect of day (***χ***^2^=25.31, p<0.001;Table S1), a handling method by day interaction (***χ***^2^=47.25, p<0.001;Table S2), a handling method by time interaction (***χ***^2^=29.66, p<0.001;Table S2), and a three-way interaction of handling method, time and day (***χ***^2^=29.42, p<0.001;Table S2). Tukey post hoc analyses revealed that these significant interactions were predominantly driven by changes across days and time in the tail handled mice. We found no evidence of a change in the time that tunnel handled mice spent interacting with the handler across days or between pre and post handling interaction tests (all p>0.05). However, when comparing changes in voluntary interaction for the tail handled mice, we found that mice interacted significantly less with the handler after being handled on day 1 (p<0.001). This was not the same across subsequent days (5 and 9). Instead tail handled mice increased the amount of time they spent interacting with the handler after handling (relative to pre-handling) but this was only evident at day 9 (day 9: p<0.001; day 5: p=0.122).

Therefore, whilst our data show a clear difference between the amount of time tail and tunnel handled mice spent interacting with the handler across all tests, there were some changes over time (see also: Hurst and West, 2010; Gouveia *et al.*, 2013; Clarkson *et al.*, 2018)

*1.2.2 Voluntary interaction tests during the contrast experiments (days 19 and 27)*

A full factorial GLMM was carried out on the time spent interacting with handler, with handling method, day and time as main factors (Table S3).

Table S3: Full statistical results for the Generalised Linear Mixed Model conducted for the percentage of time spent voluntarily interacting with the handler on days 19 and 27 in line with the contrast experiments.

| **Factor** | ***χ*^2^** | **p value** |
| --- | --- | --- |
| Handling method  Day  Time (pre or post handling)  Handling method x Day  Handling method x Time  Day x Time  Handling method x Day x Time | 52.2  1.32  3.94  1.06  10.5  0.72  0.30 | 5.014^e-13^ ***  0.251 ns  0.047 *  0.304 ns  0.001 ***  0.395 ns  0.584 ns |

As outlined in the main text (Figure 2A) clear differences were still evident between tail and tunnel handled mice in the time they spent interacting with the handler in line with the contrast experiments (***χ***^2^=52.2, p<0.001; Table S3). However, we also found other significant main effects and interactions. We found a significant interaction between handling method and time (***χ***^2^=10.5, p=0.001; Table S3) and a main effect of time overall (***χ***^2^=3.94, p=0.047; Table S3). Tukey post hoc analyses revealed that tail handled mice spent more time interacting with the handler after being handled compared to before they were handled (p<0.001), whereas the tunnel handled mice did not (p=0.833). This finding is in line with our previous work (Clarkson *et al.*, 2018) whereby we suggested that a possible explanation for this could be that they learnt the sequence of events and were more apprehensive of the handler before they had been picked up compared to after they had been picked up. There were no other significant main effects or interactions (Table S3).

*1.2.3 Open Field Test (day 33)*

In addition to measuring the frequency and duration in the centre of the open field (Figure 2B), we also measured the duration spent moving, velocity and total distance moved. We found that whilst mice from both groups spent similar amounts of time moving (Movement: t_62_=0.11, p=0.910; Figure S1A), tunnel handled mice tended to move further and faster, although this did not reach statistical significance (Distance travelled: t_60.8_=1.86, p=0.068, Figure S1B; Velocity: t_60.5_=1.76, p=0.084, Figure S1C).


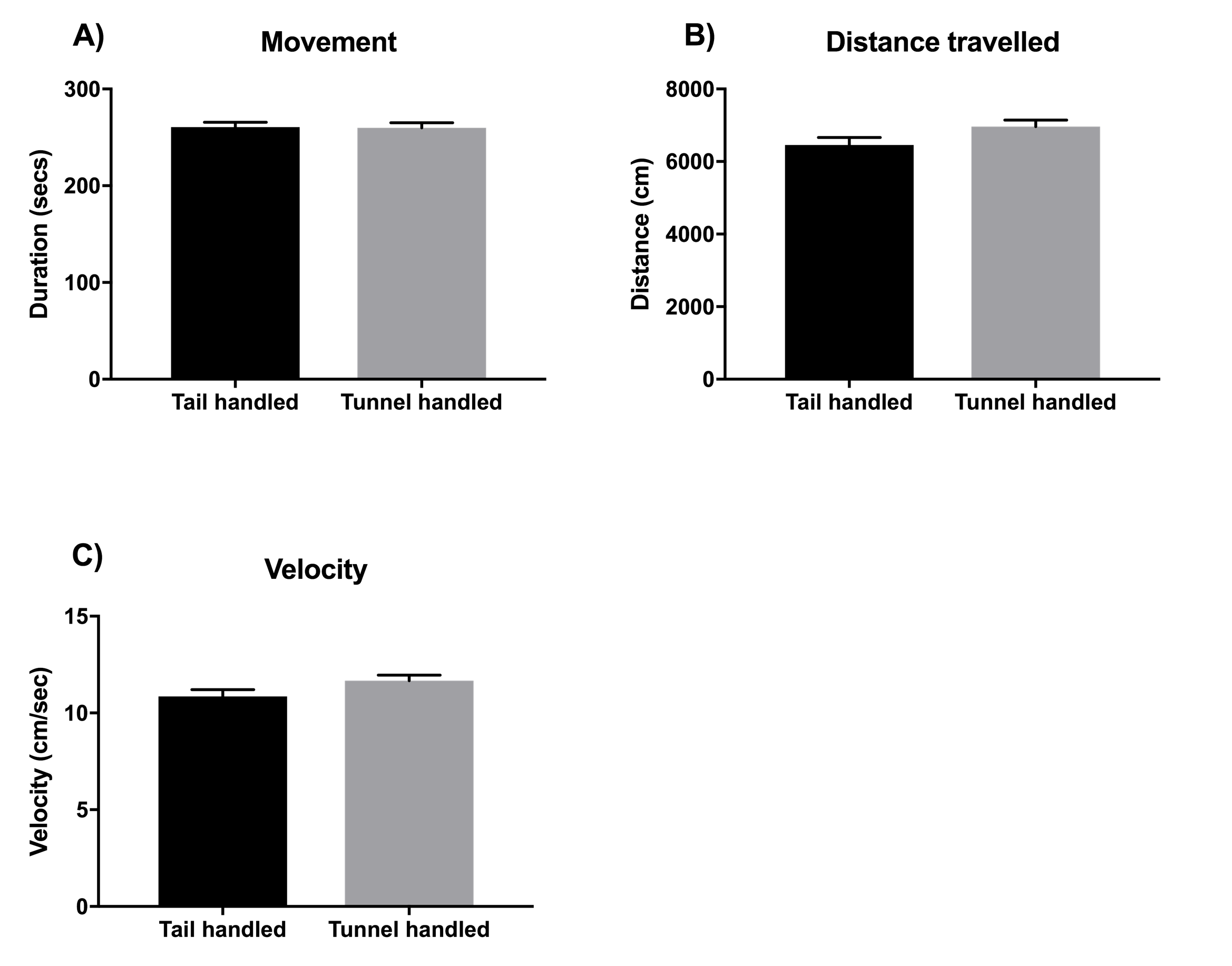


Figure S1: Additional comparisons of tail and tunnel handled mice in the open field test. A. Mean (+SEM) length of time spent moving (s). B. Mean (+SEM) distance travelled (cm). C. Mean (+SEM) velocity when moving (cm/sec).

*1.2.4 Other physiological measures (day 34)*

We found no differences in thymus weight between tail and tunnel handled mice (t_30_=1.53, p=0.136; Figure S2A), which was calculated as a percentage of their body mass in order to control for any possible differences in body mass. However, as illustrated on Figure S2B, we found no difference in bodyweight between our two handling treatments (t_30_=1.19, p=0.243).

**
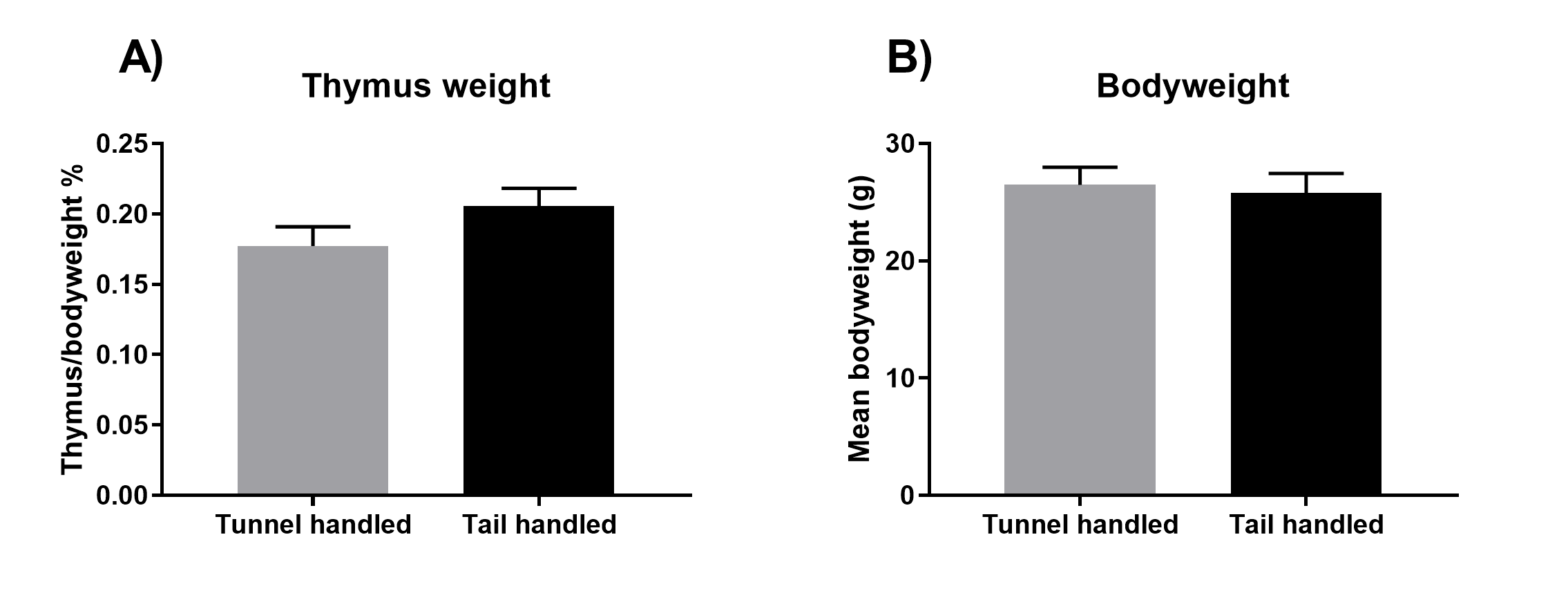
**

Figure S2: A. Mean (+SEM) thymus weight as a percentage of bodyweight for both tail and tunnel handled mice conducted on day 34. B. Mean bodyweight of tail and tunnel handled mice on day 34.

*1.2.5 Sucrose consumption (pre-shift phase: days 21-24)*

In order to determine whether the required differences in hedonic rating towards the low and high concentrations of sucrose reward were present, we analysed data from all mice at the end of the pre-shift phase. In contrast to expectations that mice should drink more of the more concentrated sucrose solution, they in fact drank significantly less of the high concentration solution compared to the low concentration solution (F_1,60_=4.39, p=0.040; Figure 2E). There was no difference in sucrose consumption between mice that were tunnel handled or tail handed (F_1,60_=1.63, p=0.207), and no significant interaction between handling and sucrose concentration (F_1,60_=0.144, p=0.706). The fact that the mice did not drink more of the higher reward meant that clear interpretation of a negative or positive contrast effect was impossible. This is because in order to test a contrast effect, the higher reward should be valued more, i.e. the mice should drink more of the high than the low reward solution. Since this did not occur, we were unable to look for contrast effects using the consumption data.

*1.2.6 Lick cluster sizes (pre-shift phase: days 21-24)*

We also analysed lick cluster sizes at the pre-shift phase to determine whether they could be used to infer the animals’ hedonic ratings of sucrose. In addition to using an interbout interval of 500ms (Figure 2E), we also tested two additional inter-bout interval criteria (250ms and 1000ms). We found that, although the results differed slightly according to the interbout interval criterion used, mice tended to have larger lick cluster sizes to the high concentration sucrose solution compared to the low concentration sucrose solution (Table S4, Figure S3). This means that lick cluster size accurately reflects the difference in hedonic value between the low and high sucrose solutions and we used it to investigate a contrast effect following a reward loss or gain. Furthermore, tail handled mice tended to have smaller lick cluster sizes, and were therefore anhedonic, compared to tunnel handled mice, confirming our affective state manipulation was effective (Table S4, Figure S3). However, we found no evidence that these factors interacted (Table S4, Figure S3). Since the inter-bout interval of 500ms provided the clearest effects for both the handling manipulation and sucrose concentration, we chose to present these data in the main text for the contrast experiments (see Figure 3).

Table S4: Statistical results for the full results of the General Linear Models conducted for lick cluster sizes according to the three interbout intervals (IBIs) for the pre-shift phase. *p<0.05 and **p<0.01.

| **Interbout Interval** | **Factor** | **F_df_** | **p value** |
| --- | --- | --- | --- |
| 250ms  500ms  1000ms | Handling method  Concentration  Handling method x Concentration  Handling method  Concentration  Handling method x Concentration  Handling method  Concentration  Handling method x Concentration | F_1,60_ = 3.14  F_1,60_ = 4.28  F_1,60_ = 0.83  F_1,60_ = 6.77  F_1,60_ = 4.07  F_1,60_ = 1.39  F_1,60_ = 9.65  F_1,60_ = 2.02  F_1,60_ = 2.19 | p=0.082 ns  p=0.043*  p=0.366 ns  p=0.012*  p=0.048*  p=0.243 ns  p=0.003**  p=0.160 ns  p=0.144 ns |


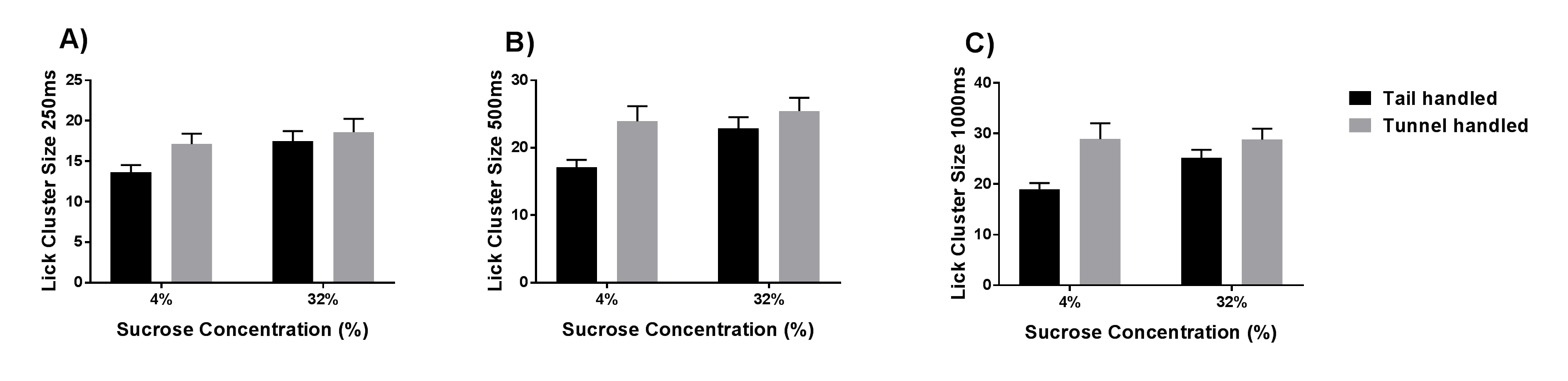


Figure S3: The mean (+SEM) lick cluster sizes for the pre-shift phase at the low (4%) and high (32%) sucrose, for tail and tunnel handled mice using three different interbout (IBI) criteria. Mean (+SEM) lick cluster size using IBI of 250ms (A), 500ms (B) and 1000ms (C).

*1.2.7 Sucrose consumption in the post-shift phases (days 25-32) for control mice only*

Since we detected no effect of handling on sucrose consumption at the pre-shift phase, we also looked to see if we could detect a difference in consumption between handling groups in the post-shift phase. Given that the shifted animals now received rewards with different hedonic values, we could only use the control mice for these analyses, since they received the same concentration of sucrose across the experiment.

In contrast to the end of the pre-shift phase, we found a significant effect of handling method on sucrose consumption across the post-shift phase. Tail handled mice drank less sucrose than tunnel handled mice at these later stages of the contrast experiments (Table S5). However, in line with the results from the pre-shift phase, we found that consumption did not accurately reflect the predicted difference in hedonic value, with mice drinking less of the more concentrated sucrose solution (Table S5).

Table S5: Full results from Linear Mixed Model for the sucrose consumption data for the control mice at the post-shift phase, including all main effects and interactions. Where *p<0.05, **p<0.01.

| **Factor** | ***χ*^2^** | **p value** |
| --- | --- | --- |
| Handling method  Sucrose concentration  Post-shift phase  Handling method x Sucrose concentration  Sucrose concentration x Post-shift phase  Handling method x Post-shift phase  Handling method x Sucrose concentration x Post-shift phase | 5.44  8.36  3.08  1.38  0.43  0.34  0.13 | 0.019*  0.0038**  0.079 ns  0.502 ns  0.805 ns  0.843 ns  0.715 ns |

**1.3 Additional analyses for the contrast experiments in the pre-shift phase**

We performed a number of additional analyses on the data from the last four days of the pre-shift phase to ensure meaningful interpretation of the subsequent contrast effect.

*1.3.1 Comparison between contrast groups with their respective controls at the pre-shift phase*

We compared the lick cluster sizes of each contrast group with their respective control group prior to the shift in sucrose consumption to help interpret the subsequent contrast effect during the post-shift phase. We calculated the mean lick cluster size for each mouse across the last four pre-shift trials four trials of and used independent samples t-tests to compare the contrast group with its control. Interestingly, only the difference was found between the tail SNC group and the tail SNC control group (t_40.7_=4.13, p<0.001). All other comparisons showed no difference in lick cluster size (tail SPC/tail SPC controls = t_53.9_=1.45, p=0.153; tunnel SNC/tunnel SNC controls = t_58.5_=0.95, p=0.347; tunnel SPC/tunnel SPC controls = t_45.9_=0.06, p=0.9557). However, as discussed in the main text, we were confident that our mice were able to detect the two rewards differently, given the concentration effect seen at the pre-shift phase when assessing responses from all mice combined with the presence of contrast effects in the post-shift phases.

*1.3.2 Stability of the control mice*

We ensured that the control mice had stabilised their lick cluster sizes during the last four pre-shift trials. We performed repeated measures ANOVAs for each control group separately and found no significant effect of trial for any of the control groups (tail SNC control: F_3,21_=0.40, p=0.757; tunnel SNC control: F_1.27,8.91_=1.22, p=0.314; tail SPC control: F_1.72,12.07_=2.96, p=0.095; tunnel SPC control: F_3,21_=2.12, p=0.128). Therefore, all control groups had stable lick cluster sizes prior to the shift in sucrose concentration and therefore enabled us to make meaningful comparisons with shifted animals.

**1.4 Results from contrast experiments according to differential Inter-bout Interval criteria**

Due to there being some debate surrounding the appropriate inter-bout interval criteria within the literature to classify licking bouts, we analysed the data across three different criteria (250, 500 and 1000ms). Although there were some quantitative differences in terms of statistical analyses for both SNC and SPC data (Tables S6-7), qualitatively the pattern of results were the same irrespective of the criterion used (Figures S4-5). We present data obtained using an inter-bout interval of 500ms in the main text for the contrast experiments given that this parameter provided the clearest effects of both handling and sucrose concentration during the pre-shift phase. Therefore, demonstrating that this parameter effectively reflected the animal’s hedonic responses towards sucrose.

**Successive Negative Contrast**


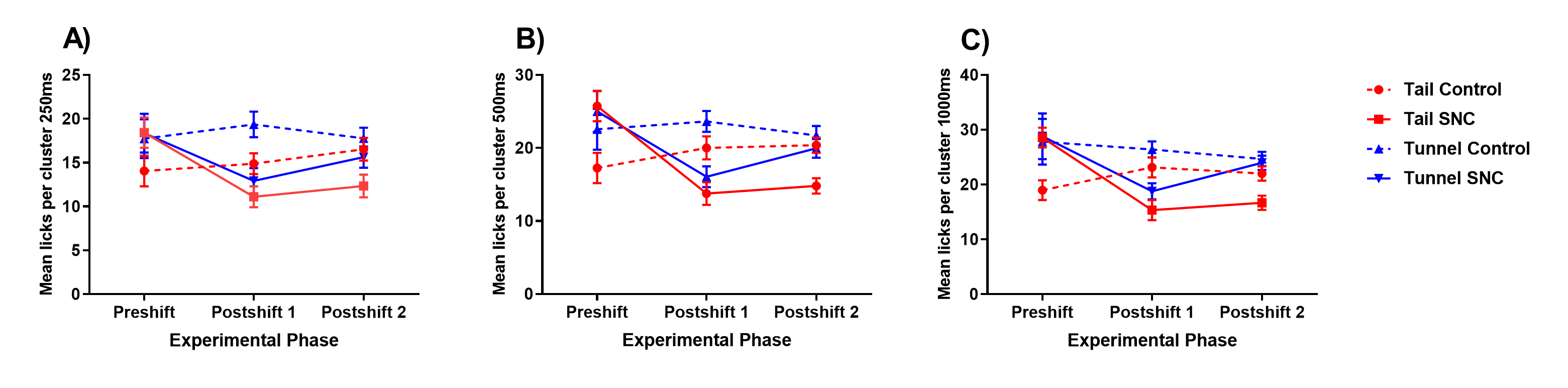


Figure S4: The mean (±SEM) lick cluster sizes for the Successive Negative Contrast (SNC) during the pre and post-shift phases, for tail and tunnel handled mice using three different interbout (IBI) criteria A. Mean (±SEM) lick cluster size using IBI of 250ms B. Mean (±SEM) lick cluster size using IBI of 500ms C. Mean (±SEM) lick cluster size using IBI of 1000ms.

Table S6: Full results from Linear Mixed Model for the Successive Negative Contrast (SNC) data, including all main effects and interactions. Where *p<0.05, **p<0.01, ***p<0.001.

| **Interbout Interval** | **Factor** | ***χ*^2^** | **p value** |
| --- | --- | --- | --- |
| 250ms  500ms  1000ms | Handling method  Contrast condition  Post-shift Phase  Handling method x contrast condition  Handling method x post-shift phase  Contrast condition x post-shift phase  Handling method x contrast condition x  post-shift phase  Handling method  Contrast condition  Post-shift Phase  Handling method x contrast condition  Handling method x post-shift phase  Contrast condition x post-shift phase  Handling method x contrast condition x  post-shift phase  Handling method  Contrast condition  Post-shift Phase  Handling method x contrast condition  Handling method x post-shift phase  Contrast condition x post-shift phase  Handling method x contrast condition x  post-shift phase | 5.87  12.54  2.62  4.96  5.32  7.36  4.92  7.56  18.37  1.71  4.44  4.15  10.50  4.10  11.07  16.70  1.51  3.49  3.63  12.59  2.38 | 0.015 *  0.0004 ***  0.105 ns  0.084 ns  0.070 ns  0.0252 *  0.0266 *  0.006 **  1.82^e-5^ ***  0.1915 ns  0.109 ns  0.126 ns  0.0053 **  0.043 *  0.0009 ***  4.38^e-5^ ***  0.219 ns  0.175 ns  0.163 ns  0.0019 **  0.123 ns |

**Successive Positive Contrast**


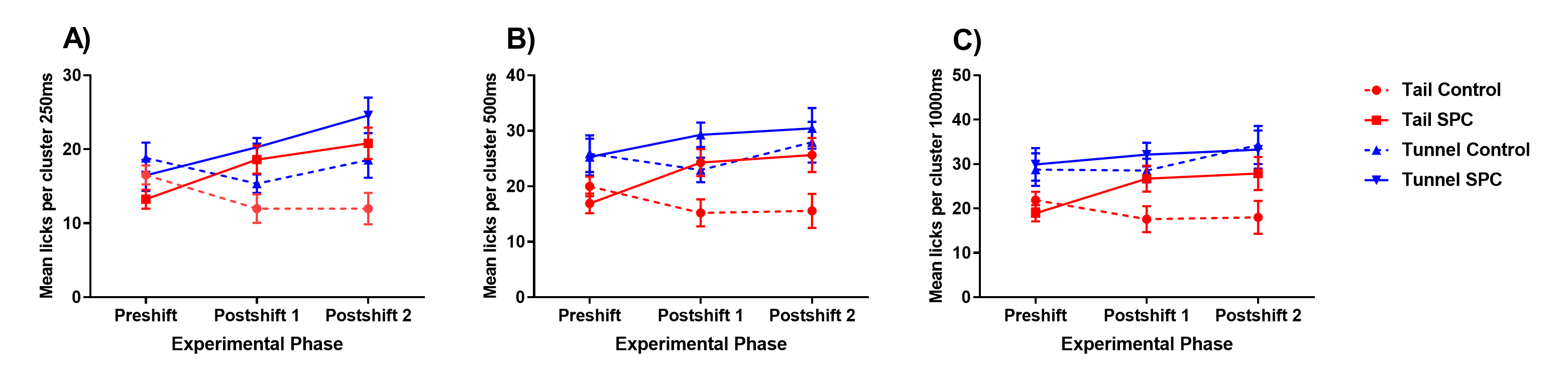


Figure S5: The mean (±SEM) lick cluster sizes for the Successive Positive Contrast (SPC) during the pre and post-shift phases, for tail and tunnel handled mice using three different interbout (IBI) criteria A. Mean (±SEM) lick cluster size using IBI of 250ms B. Mean (±SEM) lick cluster size using IBI of 500ms C. Mean (±SEM) lick cluster size using IBI of 1000ms.

| **Interbout Interval** | **Factor** | ***χ*^2^** | **p value** |
| --- | --- | --- | --- |
| 250ms  500ms  1000ms | Handling method  Contrast condition  Post-shift Phase  Handling method x contrast condition  Handling method x post-shift phase  Contrast condition x post-shift phase  Handling method x contrast condition x  Post-shift phase  Handling method  Contrast condition  Post-shift Phase  Handling method x contrast condition  Handling method x post-shift phase  Contrast condition x post-shift phase  Handling method x contrast condition x  Post-shift phase  Handling method  Contrast condition  Post-shift Phase  Handling method x contrast condition  Handling method x post-shift phase  Contrast condition x post-shift phase  Handling method x contrast condition x  Post-shift phase | 4.94  12.80  8.30  0.57  2.77  1.17  0.13  8.14  7.17  2.75  2.15  1.89  1.37  1.03  8.31  2.90  2.44  2.83  1.96  1.52  1.01 | 0.026 *  0.0003 ***  0.004 **  0.752 ns  0.250 ns  0.556 ns  0.719 ns  0.004 **  0.007 **  0.098 ns  0.342 ns  0.389 ns  0.504 ns  0.311 ns  0.0039 **  0.089 ns  0.118 ns  0.243 ns  0.376 ns  0.468 ns  0.316 ns |

Table S7: Full results from Linear Mixed Model for the Successive Positive Contrast (SPC) data, including all main effects and interactions. Where *p<0.05, **p<0.01, ***p<0.001.

**References**

Clarkson, J. M. *et al.* (2018) ‘Handling method alters the hedonic value of reward in laboratory mice’, *Scientific Reports*, 8(1), p. 2448.

Gouveia, K. *et al.* (2013) ‘Reducing Mouse Anxiety during Handling: Effect of Experience with Handling Tunnels’, *PLoS ONE*. Edited by E. M. Mintz. Public Library of Science, 8(6), p. e66401.

Hurst, J. L. and West, R. S. (2010) ‘Taming anxiety in laboratory mice’, *Nature*, 7(10), pp. 825–826.
